# Supplementary material for: Paragraph: a graph-based structural variant genotyper for short-read sequence data
Source: Genome Biol. 2019 Dec 19;20:291. doi: 10.1186/s13059-019-1909-7 (PMC6921448; doi:10.1186/s13059-019-1909-7)
Supplement: Supplementary file 2 — Additional file 2. Review history. [file 13059_2019_1909_MOESM2_ESM.docx]

Review History

**First round of review**

**Reviewer 1**

**Are you able to assess all statistics in the manuscript, including the appropriateness of statistical tests used?**

Yes, and I have assessed the statistics in my report.

**Comments to author:**

The authors introduce Paragraph, a C++/Python tool to genotype structural variations (SVs) from short-read data. As opposed to methods such as Manta, Lumpy and Delly that (also) support de novo identification of SVs, Paragraph needs to be provided with a reference database of known SVs. Paragraph builds a sequence graph structure such that each possible SV in the reference database exists in this graph as a contiguous sequence path. By re-aligning reads near potential SV breakpoints to this graph, the presence or absence of SVs is assessed. To this end, read counts supporting each allele are assumed to be Poisson-distributed.

In a first test, the recall of insertions and deletions (larger than 50 bp) is assessed for a single human individual and for various SV genotypers (Paragraph, SVTyper and Delly Genotyper) and de novo SV callers (Manta, Lumpy and Delly). The ground truth SVs were established using accurate long-read technology. In general, this feels like a dangerous benchmark setup: the SV genotypers are provided with a set of all SVs present in the sample (and only those!). Therefore, any method that would simply report all SVs present in the reference database would show a perfect recall. The benchmark setup as provided in the manuscript thus risks favouring overly aggressive genotypers. Nevertheless, the benchmark shows that existing SV genotypers (other than Paragraph) are still relatively immature: (i) SVTyper and Delly genotyper appear unable to call insertions, (ii) SVTyper underperforms for short deletions, (iii) Delly genotyper underperforms for long deletions and surprisingly performs even worse than Delly in de novo mode for long deletions.

The authors also demonstrate the need for highly accurate SV reference databases. When prior SVs include errors in their breakpoints, the recall drops rapidly with increasing breakpoint deviations. This is demonstrated only for Paragraph, the same can be assumed for other SV genotypers. The authors also show that generating an accurate SV reference database (with only few breakpoint deviations) is difficult due to the fact that many SV breakpoints occur in tandem repeats.

In a second test, the authors also assess the precision of by supplementing the prior SV dataset with SVs from a second sample. Nevertheless, the prior SV database is still tailored towards the dataset for which it is tested: according to my own calculations, it contains 22532 SVs, 17311 (76.8%) which actually exist in the test sample and only 5221 (23.2%) which do not. Again, even very aggressive methods will still show a high precision. In this reviewer's opinion, the authors should have provided a comprehensive analysis on precision and recall in a single section, using a single, well-chosen prior SV database. I can imagine that a reference database that contains all SVs that exist in the population will be much larger than what is used by the authors.

Finally, Paragraph is applied to 100 unrelated individuals. Interestingly, certain SVs occur with nearly 100% allele frequency, indicative that the reference genome sequence could be improved.

From a methodological point of view, Paragraph appears to be straightforward. It is therefore not immediately clear why Paragraph performs better on current datasets than other tools. The GSSW library should be adequately references since this is one of the main building blocks of the methods. Finally, it is not immediately clear whether or not paired-end read information is used by Paragraph.

**Reviewer 2**

**Are you able to assess all statistics in the manuscript, including the appropriateness of statistical tests used?**

Yes, and I have assessed the statistics in my report.

**Comments to author:**

Chen et al. reported a novel algorithm to do genotyping of structural variants (SV), Paragraph, which is based on graph construction of the reads aligned to target SV regions. The authors have evaluated the performance of Paragraph using HG002 short read set by the HG002 genotyped SV dataset (LRGT) as a reference, which has been constructed with PacBio CCS read data from GIAB. The method is also applied to low coverage CLR (contiguous long reads) data. The description is clear, with an comparison of accuracy measurement with known SV callers attached which is helpful for the readers to evaluate its performance.

However, there are some points needed to be addressed, this reviewer thinks.

1. The authors compared the performance of Paragraph with SVTyper, Delly, manta, and Lumpy. This reviewer thinks that BayesTyper (Sibbesen et. Al., "Accurate genotyping across variant classes and lengths using variant graphs" Nat Genet 50, 1054-1059, 2018) needed to be compared. BayesTyper calls genotypes of variants using variant graphs and Bayesian approach, which is similar to the method used in Paragraph, although there is a difference in using read k-mer and read sequences for graph construction between the two studies. Thus, the authors should clearly explain the differences of the methods between BayesTyper and Paragraph and describe the advantage of Paragraph that can be given by the differences.

2. There are also other two papers needed to be cited, and it is also helpful for the readers if comparisons are available in this manuscript:

- Hickey et. al., "Genotyping structural variants in pangenome graphs using the vg toolkit" bioRxiv 2019

- Antaki et al., "SV2: accurate structural variation genotyping and de novo mutation detection from whole genomes" Bioinformatics 15, 1774-1777, 2018

3. The authors regard recall as the fraction of SVs genotyped as "non-reference" (page 28, line 6). Does this mean that non-reference heterozygote and homozygote are similarly counted? This strategy is used in de novo SV callers, but as for SV genotypers this reviewer thinks it is not appropriate. Please clarify, and please count consistent genotypes for recall calculation.

Minor points

1. The last term in formula 5 (page 24) might be $\lambda \epsilon$

2. Supplementary Table 2 is not cited from the main text. This would be the description of HG002_SVs_Tier1_v0.6.vcf ? However, number of non-ref DEL and INS are 8287 and 11479 respectively, which is different from the numbers in this table. Please clarify.

**Authors Response**

**Point-by-point responses to the reviewers’ comments:**

Reviewer #1:

The authors introduce Paragraph, a C++/Python tool to genotype structural variations (SVs) from short-read data. As opposed to methods such as Manta, Lumpy and Delly that (also) support de novo identification of SVs, Paragraph needs to be provided with a reference database of known SVs. Paragraph builds a sequence graph structure such that each possible SV in the reference database exists in this graph as a contiguous sequence path. By re-aligning reads near potential SV breakpoints to this graph, the presence or absence of SVs is assessed. To this end, read counts supporting each allele are assumed to be Poisson-distributed.

In a first test, the recall of insertions and deletions (larger than 50 bp) is assessed for a single human individual and for various SV genotypers (Paragraph, SVTyper and Delly Genotyper) and de novo SV callers (Manta, Lumpy and Delly).

The ground truth SVs were established using accurate long-read technology. In general, this feels like a dangerous benchmark setup: the SV genotypers are provided with a set of all SVs present in the sample (and only those!). Therefore, any method that would simply report all SVs present in the reference database would show a perfect recall. The benchmark setup as provided in the manuscript thus risks favouring overly aggressive genotypers. Nevertheless, the benchmark shows that existing SV genotypers (other than Paragraph) are still relatively immature: (i) SVTyper and Delly genotyper appear unable to call insertions, (ii) SVTyper underperforms for short deletions, (iii) Delly genotyper underperforms for long deletions and surprisingly performs even worse than Delly in de novo mode for long deletions.

The authors also demonstrate the need for highly accurate SV reference databases. When prior SVs include errors in their breakpoints, the recall drops rapidly with increasing breakpoint deviations. This is demonstrated only for Paragraph, the same can be assumed for other SV genotypers. The authors also show that generating an accurate SV reference database (with only few breakpoint deviations) is difficult due to the fact that many SV breakpoints occur in tandem repeats.

In a second test, the authors also assess the precision of by supplementing the prior SV dataset with SVs from a second sample. Nevertheless, the prior SV database is still tailored towards the dataset for which it is tested: according to my own calculations, it contains 22532 SVs, 17311 (76.8%) which actually exist in the test sample and only 5221 (23.2%) which do not. Again, even very aggressive methods will still show a high precision. In this reviewer's opinion, the authors should have provided a comprehensive analysis on precision and recall in a single section, using a single, well-chosen prior SV database. I can imagine that a reference database that contains all SVs that exist in the population will be much larger than what is used by the authors.

Finally, Paragraph is applied to 100 unrelated individuals. Interestingly, certain SVs occur with nearly 100% allele frequency, indicative that the reference genome sequence could be improved.

*The reviewer highlights several very important deficiencies in our analysis and we thank him/her for these suggestions. We have made changes that we believe now significantly improve the short-comings of our initial analysis and describe these changes below. Most substantially we revised the assessment of accuracy using a much larger control database of SVs collected from 3 samples sequenced with PacBio CCS data. This allows for a much more comprehensive assessment of the accuracy of both false positives and false negative variants. Below we outline the major revisions we have made:*

*We agree with the reviewer that the initial study design created the possibility that a very aggressive method could perform better because of the high number of TPs relative to TNs. Because we have found accurate breakpoints to be a major confounder to accurate genotyping, we focussed the main points of our analysis to samples with high-depth CCS data. Since we started that initial analysis, high-depth CCS sequence data for two additional samples has become available from the Genome-In-A-Bottle consortium (GIAB) and so we have repeated our analysis using SV calls from all three samples. This allows for a much more balanced assessment of variants, and we now test about the same number of TNs as TPs. This has impacted two parts of the manuscript and we have changed our description of the benchmarking data as described in the Results - Construction of long read-based ground truth on pages 7 and 8 and the Methods - Long read ground truth and performance evaluation sections on pages 28 and 29.*

*Based on this new high quality catalog of SVs, we repeated our comparison assessing both precision and recall simultaneously and we still show that Paragraph has the highest performance based on F-score across all variant types. For clarity, we removed the separate precision subsection entirely and have integrated the analysis of both recall and precision within the Results - Test for recall and precision on pages 8-12.*

*Additionally, by now utilizing high-quality SV calls from multiple samples we are able to better understand regions where the targeted genotypers may have difficulties. In addition to discussing the problems with SVs due to tandem repeats (TR) status, we can also categorize SVs according to the presence of nearby but different SVs and highlight that, as expected, Paragraph performs well for isolated single SVs but relatively poorly for the clustered SVs, as do all tested methods. This highlights a limitation of both our method and the other methods and on page 22 (lines 4-6) we highlight that future work will work to allowing Paragraph to call multiple nearby SVs.*

From a methodological point of view, Paragraph appears to be straightforward. It is therefore not immediately clear why Paragraph performs better on current datasets than other tools. The GSSW library should be adequately referenced this is one of the main building blocks of the methods. Finally, it is not immediately clear whether or not paired-end read information is used by Paragraph.

*While it is beyond the scope of this study to quantify specific limitations in other methods that may have led to poorer performance, we do feel that these performance differences are likely driven by two mechanisms: 1) by extracting and graph aligning all of the reads in the extended regions around the target SVs, Paragraph may be recovering mis-aligned reads better or 2) Paragraph may be doing a better job of aligning reads with high-error rates and filtering alignment artifacts. We have observed that reads may be significantly lower quality in low complexity regions and our parameters may work better to extract information from low quality reads. Relatedly, see our response to Reviewer 2 about our results using BayesTyper.*

*Thanks for pointing out our failure to reference the GSSW library and now we reference that on page 23 (second to last line).*

*We currently do not use paired-end read information and we have made that limitation explicit on page 23 (last line). We feel that this is an area where Paragraph could improve in the future and have plans to quantify the improvement that can be obtained by using the read pair information and implement read pair information in future releases of Paragraph.*

Reviewer #2

Chen et al. reported a novel algorithm to do genotyping of structural variants (SV), Paragraph, which is based on graph construction of the reads aligned to target SV regions. The authors have evaluated the performance of Paragraph using HG002 short read set by the HG002 genotyped SV dataset (LRGT) as a reference, which has been constructed with PacBio CCS read data from GIAB. The method is also applied to low coverage CLR (contiguous long reads) data. The description is clear, with a comparison of accuracy measurement with known SV callers attached which is helpful for the readers to evaluate its performance.

However, there are some points needed to be addressed, this reviewer thinks.

1. The authors compared the performance of Paragraph with SVTyper, Delly, manta, and Lumpy. This reviewer thinks that BayesTyper (Sibbesen et. Al., "Accurate genotyping across variant classes and lengths using variant graphs" Nat Genet 50, 1054-1059, 2018) needed to be compared. BayesTyper calls genotypes of variants using variant graphs and Bayesian approach, which is similar to the method used in Paragraph, although there is a difference in using read k-mer and read sequences for graph construction between the two studies. Thus, the authors should clearly explain the differences of the methods between BayesTyper and Paragraph and describe the advantage of Paragraph that can be given by the differences.

*We have referenced BayesTyper and ran BayesTyper on our evaluation data for comparison. We expected and observed that BayesTyper would produce poorer results than Paragraph because it relies on exact matching k-mers which requires exact breakpoints and/or insertion sequences whereas Paragraph can align with gaps. We also expect the elevated error rates in these regions will be problematic to BayesTyper because low complexity regions are known to also have high error rates in most sequencers. When we ran BayesTyper on NA24385 (HG002) with default parameters the recall and precision were 0.47 and 0.94, respectively. This is primarily because BayesTyper failed to compute a genotype call for over half (56%; 11,396) of these SVs in our LRGT. We have described these results on Page 10 (lines 4-9) but because of the high no call rate we did not include this method in our full analysis.*

2. There are also other two papers needed to be cited, and it is also helpful for the readers if comparisons are available in this manuscript:

- Hickey et. al., "Genotyping structural variants in pangenome graphs using the vg toolkit" bioRxiv 2019

- Antaki et al., "SV2: accurate structural variation genotyping and de novo mutation detection from whole genomes" Bioinformatics 15, 1774-1777, 2018

*We have cited these manuscripts and now discuss the differences between them and our method in the introduction. Compared to the BayesTyper referenced above, both of these methods require additional analysis such as a training data set (SV2) or a whole genome realignment step (vg). These additional steps make them more difficult for users to ask simple questions such as “do the samples in my study have a given SV and, if so, which ones”. We have highlighted these differences and the different use cases for our method on Page 4 (paragraph 2) and Page 21 (last paragraph).*

3. The authors regard recall as the fraction of SVs genotyped as "non-reference" (page 28, line 6). Does this mean that non-reference heterozygote and homozygote are similarly counted? This strategy is used in de novo SV callers, but as for SV genotypers this reviewer thinks it is not appropriate. Please clarify, and please count consistent genotypes for recall calculation.

*We have not compared genotypes in the main text because long read SV callers may not accurately genotype the variants identified. We have highlighted this logic in the text but have also calculated the genotype concordance and included that in our supplementary methods. Our result shows that Paragraph and the long read caller (PBSV) genotypes are 81% concordant in deletions and 72% concordant in insertions. As a comparison, Delly Genotyper and Lumpy are both 73% concordant with PBSV genotypes in deletions. We mention this on page 9 (lines 2-3).*

*We think that it is important to note that we also performed an HWE assessment in the population to assess overall GT accuracy. In the absence of selection, genotypes should follow the HW expectation. In general, users may just have information on a particular SV in the population without having specific truth data for assessing genotyping accuracy. In these cases, QC methods such as HWE assessment will allow users to filter down to a subset of highly accurate variants and work towards improving the methods for the other variants. We think the HWE assessment in the manuscript has provided a sense of how accurate Paragraph genotype is independent of possible confounding effects of the genotyping performance of long read caller.*

Minor points

1. The last term in formula 5 (page 24) might be $\lambda \epsilon$

*Thanks for pointing that typo out. We have corrected this formula.*

2. Supplementary Table 2 is not cited in the main text. This would be the description of HG002_SVs_Tier1_v0.6.vcf ? However, a number of non-ref DEL and INS are 8287 and 11479 respectively, which is different from the numbers in this table. Please clarify.

*That table was placed there in error and we have removed it and apologize for the confusion.*

**Second round of review**

**Reviewer 1**

The authors have adequately addressed all of my comments and I feel the manuscript can now be accepted as is.
